# Identification and estimation of lodging in bread wheat genotypes using machine learning predictive algorithms

**DOI:** 10.1186/s13007-023-01088-w

**Published:** 2023-10-17

**Authors:** Ehsan Rabieyan, Reza Darvishzadeh, Hadi Alipour

**Affiliations:** Department of Plant Production and Genetics, Urmia University, Urmia, Iran

**Keywords:** Image processing, Lodging, Machine learning, Random forest, Wheat

## Abstract

**Background:**

Lodging or stem bending decreases wheat yield quality and quantity. Thus, the traits reflected in early lodging wheat are helpful for early monitoring to some extent. In order to identify the superior genotypes and compare multiple linear regression (MLR) with support vector regression (SVR), artificial neural network (ANN), and random forest regression (RF) for predicting lodging in Iranian wheat accessions, a total of 228 wheat accessions were cultivated under field conditions in an alpha-lattice experiment, randomized incomplete block design, with two replications in two cropping seasons (2018–2019 and 2019–2020). To measure traits, a total of 20 plants were isolated from each plot and were measured using image processing.

**Results:**

The lodging score index (LS) had the highest positive correlation with plant height (r = 0.78**), Number of nodes (r = 0.71**), and internode length 1 (r = 0.70**). Genotypes were classified into four groups based on heat map output. The most lodging-resistant genotypes showed a lodging index of zero or close to zero. The findings revealed that the RF algorithm provided a more accurate estimate (R^2^ = 0.887 and RMSE = 0.091 for training data and R^2^ = 0.768 and RMSE = 0.124 for testing data) of wheat lodging than the ANN and SVR algorithms, and its robustness was as good as ANN but better than SVR.

**Conclusion:**

Overall, it seems that the RF model can provide a helpful predictive and exploratory tool to estimate wheat lodging in the field. This work can contribute to the adoption of managerial approaches for precise and non-destructive monitoring of lodging.

## Background

Lodging is defined as the displacement of the root anchorage and/or the irreversible bending of crop stems from the vertical [1]. This situation causes some difficulties, including raised drying costs, slowed harvest, reduced grain quality, destructed canopy structure, and drastic yield losses of up to 85% [2–4]. Lodging in crops is derived from the complicated interactions between agronomical, environmental, and genetic factors, making this event distinctive with various onset, intensity, and duration [5]. A complex genetic architecture underlies wheat lodging physiology [6–8]. A handful of small to moderate effect quantitative trait loci (QTL) have been identified, accounting for 2–27% of stem strength and lodging variation [9–11]. The evaluation of lodging level is challenging because of the absence of data associated with it, the lack of standard scales to present it, the random distribution of lodging on a farm, and complicated interactions between genetic and environmental elements [12–14]. As a main challenge, there aren’t annual statistics of lodged areas related to various crops at global, regional, or local scales [15].

Crop agronomists and physiologists study lodging widely but their scope is often restricted. This includes agronomic practices (that can decrease lodging-related risks), breeding programs (that can produce lodging-tolerant cultivars), [16], phenotypic studies [16], and lodging angle on wheat growth [17]. The findings of these researches indicated that three key elements determine the level of lodging and the percentage of yield loss- the lodging angle or crop angle of inclination (CAI), the spatial extent of lodging, and the crop growth stage (time of lodging incidence) [17]. By definition, CAI is known as the angles made by stems respecting the vertical situation [18]. At the time of the lodging process, a crop can undergo a sequence of steps (i.e., lodging stages) beginning with CAI∼0° (a low deviation from the vertical situation) and finishing with CAI∼90° (crop bending close to the horizontal situation) [13]. Thus, CAI levels (ranging from moderate to very severe) can be used as a critical parameter to elucidate the lodging stage and/or the canopy structure of lodged crops [16, 19].

A precise calculation of CAI contributes to the approximation of lodging-originated yield loss in crops [20]. Fischer and Stapper [21], for instance, exhibited that the wheat yield loss at a CAI of 80° was approximately threefold than that at a CAI of 45°. Lodging area percentage combined with CAI can help dedicate lodging score to a crop. i.e., a lodging severity indicator combining lodged area and CAI [4]. An estimation of CAI can therefore be useful to insurance loss estimators (to get a view of the damage level) and farmers (to keep down the harvesting loss) [22]. The classic methodologies to evaluate lodging stages and calculate CAI rely on visual rating and manual tools. These methodologies are costly and time consuming, therefore, their usage is severely restricted for covering large regions. Moreover, the high spatial variation related to lodging makes it difficult to capture this diversity with manual tools [1]. As an alternative approach, machine learning predictive approaches have attracted a lot of attention in the crop academic community to meet the challenges of classical methodologies in lodging assessment [13].

The digital analysis offers a promising method for examining morphological discrepancies from an ecological, taxonomic, and phylogenetic perspective [23]. Through imaging-based phenotypic evaluations, a range of agronomic traits has been discovered and translated, providing a better understanding of the relationship between important breeding traits [24]. The Chauhan et al. [17] study found that the use of synthetic aperture radar data for lodging assessment was only mentioned in eight peer-reviewed articles publications between 1984 and 2018, four of which used satellite-based remote sensing data. Most studies examined the behavior of remote sensing signals in relation to lodged crops (primarily for detection purposes). Meanwhile, this method is only suitable for large cultivated areas [17]. However, image processing technology has been identified as a candidate tool for crop phenotyping detection in recent decades, which can be used for small cultivated areas (< 10 m^2^).

Estimation of crop traits from field data has been carried out by various modeling procedures, such as support vector regression (SVR), artificial neural network (ANN), random forest regression (RF), and multiple linear regression (MLR) [22, 25–31]. Although these approaches are sound theoretically in data interpretation, the inversion of crop traits is still challenging because of intensive data requirements, restricted operational usage, and inherent complexity [32, 33]. There is no report, to the best of our knowledge, on the application of regression and machine learning predictive models for the estimation of lodging in Iranian wheat accessions. Thus, the purpose of this paper is to screen the wheat genotypes for lodging resistance and compare the efficiency of MLR, ANN, SVR, and RF for predicting lodging and its related traits.

## Materials and methods

### The research area

**Fig. 1:**
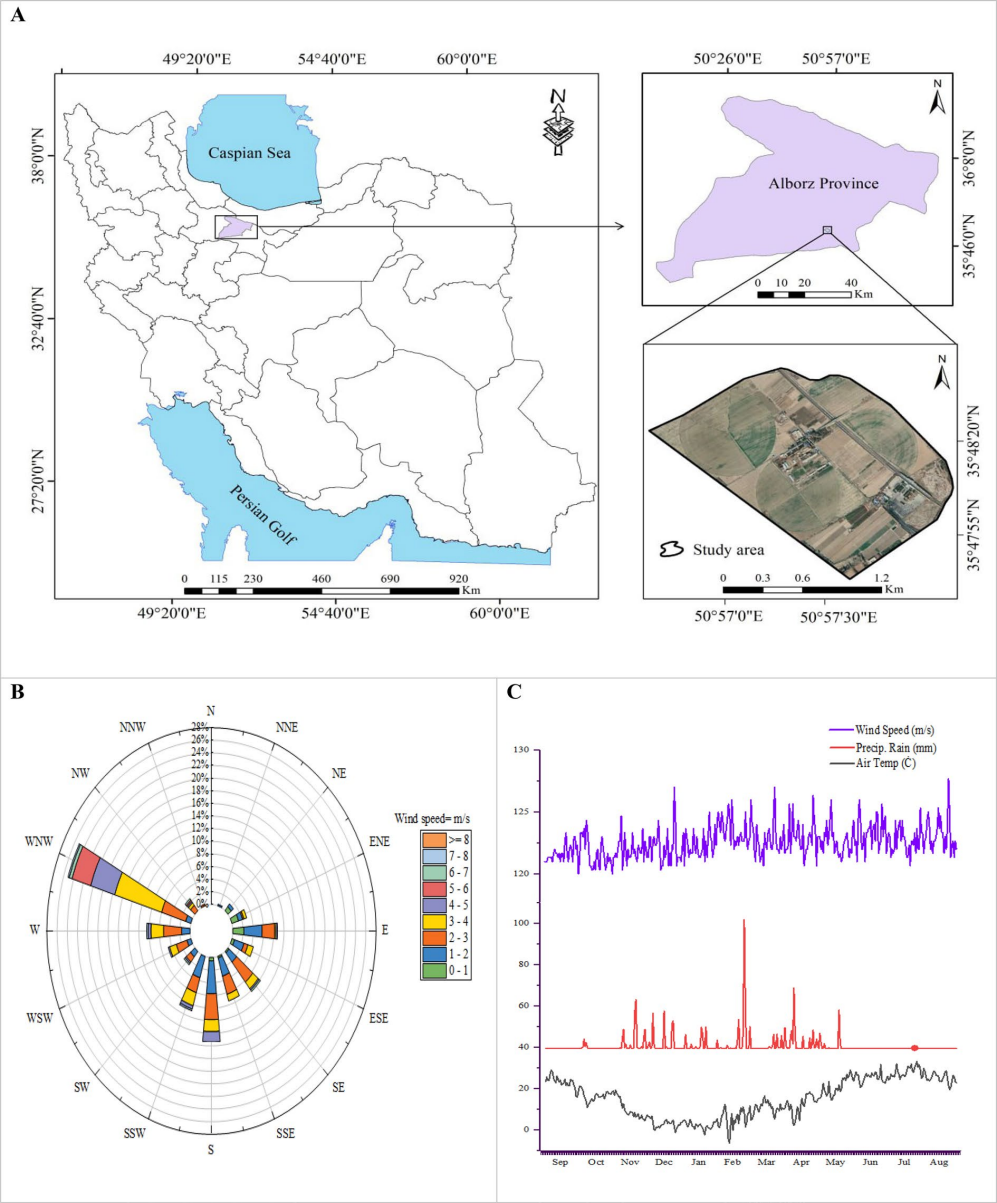
The geographical location of the study area (**A**), and average 2 year wind rose plot (**B**) and climatic parameters (**C**)

This research was performed in the research field of the Agriculture & Natural Resources Campus (35°48′59″N, 51°58′48″E, 1321 m elevation), located in the province of Alborz, Iran (Fig. 1). The study area is shown in Fig. 1A, the wind rose plot in Fig. 1B, and the climatic characteristics in Fig. 1C. This field covers around 246 ha and its main crops are wheat, corn, barley, and alfalfa. The climate in this area is dry and warm. The soil texture mainly consisted of clay and silt. The annual average temperature and precipitation are 22 °C and 248 mm, respectively. The chemical and physical characteristics of the field soil are given in Table 1.

**Table 1:** Some physical and chemical soil properties of the wheat field

Years	Depth (cm)	Available K (mg kg^−1^)	Available P (mg kg^−1^)	pH	EC (dS m^−1^)	Soil texture	Sand (%)	Silt (%)	Clay (%)	OC (%)	Total N (%)
2018–2019	0–30	125	8.3	8.4	0.97	Clay loam	25	44	31	0.76	0.09
	30–60	125	2.2	8.5	1.16	Clay loam	26	44	30	0.62	0.07
2019–2020	0–30	127	9.1	8.3	0.93	Clay loam	24	45	31	0.79	0.09
	30–60	122	3.2	8.4	1.03	Clay loam	26	43	31	0.67	0.07

### Experimental design

To evaluate wheat lodging and related traits under normal conditions, a total of 228 wheat accessions (156 native landraces and 72 cultivars) were tested in an alpha-lattice experiment, randomized incomplete block design, with two replications in two cropping seasons (2018–2019 and 2019–2020). The sizes of the plots were adjusted to 2 m^2^. To measure lodging and related traits in wheat accessions, notes were taken in the pre-physiological stage.

### Trait measurements

The traits measured in this study were as follows: Grain yield (GY, gr per plant), spike area (SA, cm^2^), spike weight (SW, gr), days to maturity (DTM), days to flowering (DTF), days to heading (DTH), internode diameter 1 and 2 (ID1 and ID2, mm), penultimate diameter (PeD, mm), peduncle diameter (PD, mm), internode length 1 and 2 (IL1 and IL2, cm), penultimate length (Pel, cm), peduncle length (PL, cm), number of nodes (NFN), plant height (PH, cm), lodged area (LA, %), lodging score index (LS), and crop angle of inclination (CAI).

### Traditional method

**Fig. 2:**
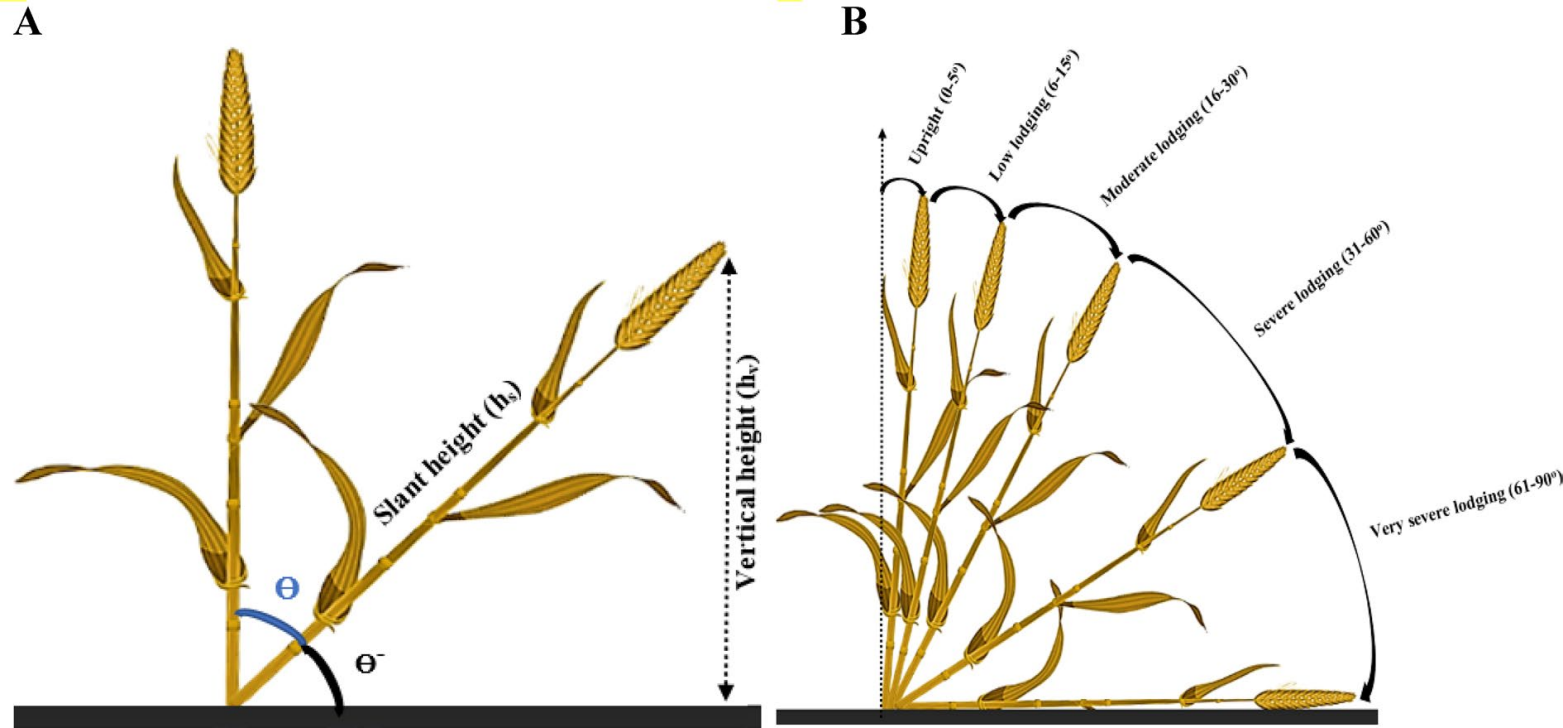
Measurement of crop angle of inclination (**A**) and presentation of various lodging stages (**B**)

To determine whether the wheat plots were lodged (L) or healthy (H) in the field, the CAI was measured from the lodged area (LA [0–100%]) and the vertical (CAI [0–90°]) in each plot (Fig. 2A, B) [32, 33]. CAI was measured by a plumb bob and trigonometric computing. The string of the plumb bob was suspended from the top of the crop and when the tip of the plumb came in contact with the soil, an accurate calculation of the vertical height (hv) was possible. To determine the slant height (hsl), a plumb bob was used for lodged plants. CAI was then estimated from the vertical via Eq. (1):

1 $$\theta degree={90}^{\mathrm{o}}-{Sin}^{-1}\frac{{h}_{v}}{{h}_{sl}}$$

where *hv* is the vertical height, and *hv* is the slant height.

**Fig. 3:**
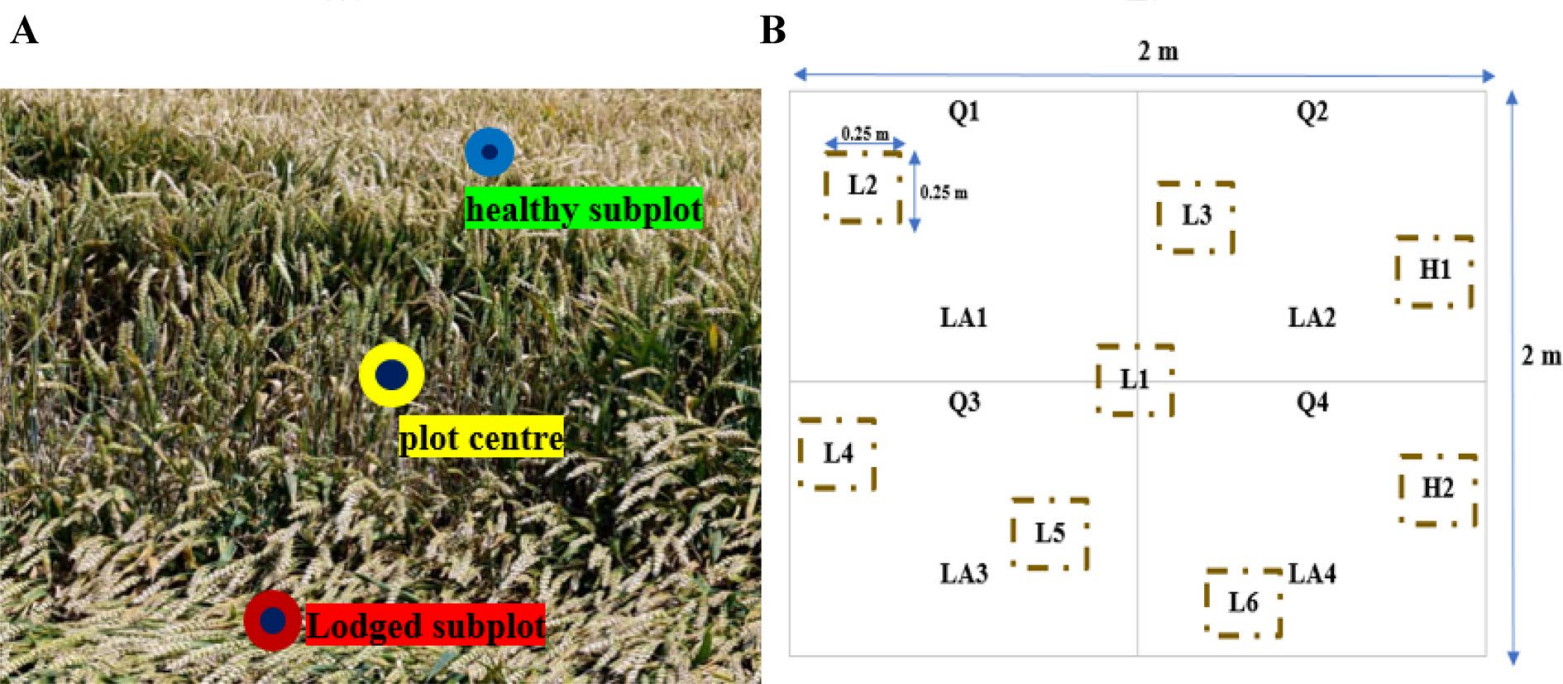
Presentation of the plot center and the healthy/lodged subplots in the field (**A**). Division of the plot into four quadrants Q1, Q2, Q3, and Q4 (**B**). LA1, LA2, LA3, and LA4 are corresponding to the lodged area in each quadrant. In this scenario, H1 and H2 present the healthy subplots while L1 to L6 are the lodged subplots. The CAI is estimated via averaging the CAI and LA calculated in the six lodged subplots and in each quadrant, respectively

LA was also evaluated visually by a quadrant methodology. In this approach, the LA % was examined in each of the four quadrants from the center of each plot and then sum to achieve the final LA for the plots. Figure 3A, B depict lodged and healthy subplots. In healthy plots, the traits were measured in three subplots (0.25 m^2^) whereas, for lodged plots, the number of subplots was increased to 4–8 for accounting for spatial heterogeneity in each lodged patch.

A normalized lodging score index (LS [0–1]) was estimated that merges LA and CAI for defining the severity classes of lodging and healthy (Fig. 3B and Eq. 2). The plot was labeled as H (LS = 0.0) if no lodging was observed. In the presence of lodging, wheat plots were classed as very severely lodged (VSL) (0.61 < LS ≤ 1.0), severely lodged (SL) (0.31 < LS ≤ 0.60), moderately lodged (ML) (0.16 < LS ≤ 0.30), low lodged (LL) (0.06 < LS ≤ 0.15) and Upright (0.0 < LS ≤ 0.05).

2 $$LS=\frac{LA}{100}\times \frac{CAL}{{90}^{\mathrm{o}}}$$

where *LS* is the lodging score index, *LA* is the lodged area, and *CAI* is the crop angle of inclination.

To measure other traits, a total of 20 plants were isolated from each plot. Traits PH, NFN, IL1, IL2, ID1, ID2, and SA were measured by using a digital caliper.

### Image processing

A basic handheld phenocart was equipped with a Canon SX540HS camera. The phenocart stood 2.1 m tall. The phenocart was equipped with a 1 m long L-shaped metal rod. The open-lens camera was 2 m above the ground and positioned on an inverted L-shaped metal pole. The images were captured during the pre-physiological stage. In addition, images were captured with the camera’s Scene Intelligent Auto mode for two consecutive days from 10:00 AM to 2:00 PM. when the sky was entirely sunny. Consequently, no color correction was made to the photographs that were taken. To have consistent illumination, the flash function was also disabled. All photos are taken in RGB and are stored in the 3240 × 4320 pixels JPEG format. Machine learning models frequently employ photos [34].

A function for color threshold based on CIELAB color space (L × a × b) was defined in Python 3.7 software. Cropped RGB images were converted to L × a × b color space. The first channel, L, which runs from black (0) to white (+ 100), was left alone, while the second channel, a, which runs from green (− 100) to red (+ 100), was cut in half and defined from 0 to + 100, and the third channel, b, which runs from blue (-100) to yellow (+ 100), was similarly cut in half and defined from 0 to + 100. The masking images were converted to binary format. The black pixels of the cool color range (from low light to dark green and blue) and the white pixels of the warm color range (from low light to dark red and yellow) are served by this strategy [34]. Finally, for each design, the black-to-white color ratio was calculated and saved in a text file as an indication of the lodged area (LA).

**Fig. 4:**
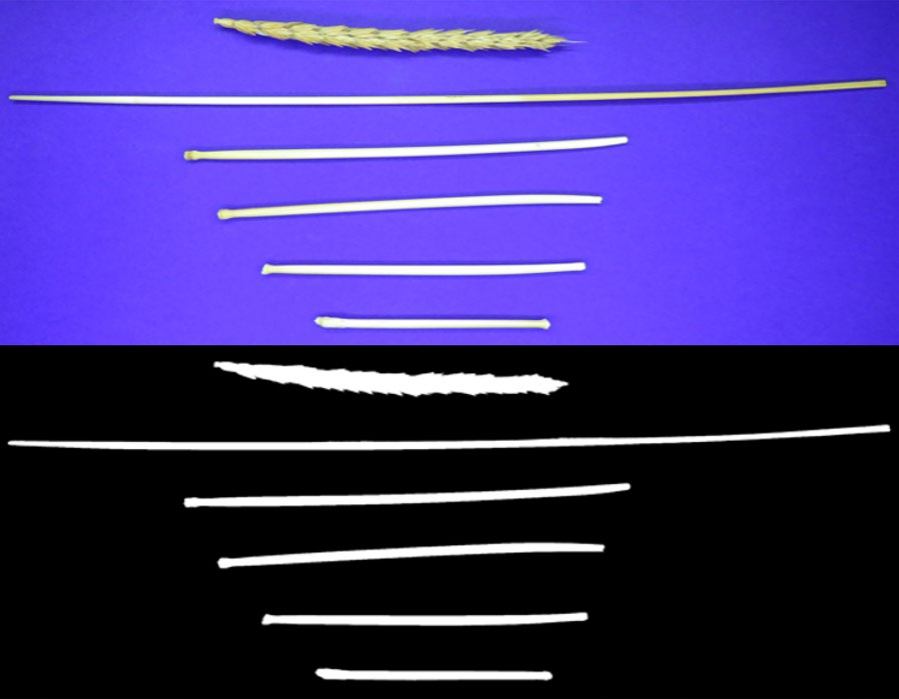
Graphical illustration of morphology traits measured in wheat plants

To measure other traits based on image processing, a total of 20 plants were isolated from each plot and they were divided into components according to Fig. 4 from the location of the node. Traits PH, NFN, IL1, IL2, ID1, ID2, and SA were measured by using image processing. For this purpose, inspired by the modified method of Leon et al. [35], a wooden box was made with dimensions 50 × 50 cm, height 60 cm, thickness of 16 mm, with 5 floors, and a distance of 10 cm between them. The floors were separated by a square wooden plate 46 cm in length. The camera was mounted on a styrofoam base at a 90° angle. A filament LED was used to create the light and installed at a 45° angle to the camera. The inner surface of the box was completely covered with black Fabriano Paperboard to prevent light reflection. The samples were placed at a distance of 10 cm from the Canon SX540HS camera lens with a resolution of 25 megapixels having the following settings: sensor’s sensitivity to light (ISO): 400; shutter speed: 1.60; aperture: f 4; flash: Off; zoom: no zoom. Python 3.7 was utilized to calculate a total of 11 variables of wheat lodging (Fig. 4) [23, 36].

To measure the trait SW, a total of 20 spikes were selected and then weighed, and their mean was reported as SW. After completing the drying process of the spikes at 70 °C for 48 h and separating the straw from the spikes, the GY of a single plant was calculated. To measure phenological traits, DTH (50% of plot spikes appeared), DTF (50% of plants are in the flowering stage), and DTM were calculated. All measurements were taken using standard CIMMYT protocols [37].

### Machine learning approaches

To predict the LS by input traits other than LA and CAI, random forest regression (RF), support vector machine (SVM), artificial neural networks (ANNs), and multiple linear regression (MLR) were used as described by Wang et al. [38]. For this purpose, the experimental data were divided into two parts, 75% for training and 25% for testing. The characteristics of experimental data are specified in Table 2. To compare the performance of various modeling algorithms, several values including root mean square error (RMSE, Eq. 3), normalized root mean square error (nRMSE, Eq. 4), mean absolute error (MAE, Eq. 5), and determination coefficient (R^2^, Eq. 6), were estimated by using the testing data set. The predictive performance of RF, SVR, ANN, and MLR methods depends on the adjustment of the optimal values of user-defined parameters. To find the optimal value of different user-defined parameters, a large number of trials were conducted using a variety of machine learning algorithms to compare the values of [root mean square error (RMSE), relative absolute error (RAE), mean absolute error (MAE), root relative square error (RRSE), and correlation coefficient (CC)] with test datasets. Therefore, these optimal values for our data set were provided in Table 3.

3 $$RMSE=\sqrt{\frac{{\sum }_{i=1}^{n}{\left({O}_{i}-{P}_{i}\right)}^{2}}{n}}$$

4 $$nRMSE=\left(\frac{RMSE}{{X}_{max}-{X}_{min} or mean}\right)*100$$

5 $$MAE=\frac{1}{n}{\sum }_{i=1}^{n}\left|{O}_{i}-{P}_{i}\right|$$

6 $${R}^{2}=\frac{{\sum }_{i=1}^{n}\left({O}_{i}-\overline{O }\right)-\left({P}_{i}-\overline{P }\right)}{\sqrt{{\sum }_{i=1}^{n}{\left({O}_{i}-\overline{O }\right)}^{2}-{\left({P}_{i}-\overline{P }\right)}^{2}}}$$

where *n* is the number of data, *O*_*i*_ is the observed values, *P*_*i*_ is the predicted values, *X*_*max*_ is the maximum data, *X*_*min*_ is the minimum data, and the bar denotes the mean of the feature.

**Table 2:** Characteristics of the training and testing data set

Variables	Input parameters	Training data				Testing data			
		Min	Max	Mean	St. dev	Min	Max	Mean	St. dev
Independent	PH	73.6	127.4	102.225	12.952	72.6	122.4	102.542	12.039
	PeD	2.24	5.65	4.02	0.592	2.45	5.34	4.052	0.598
	IL1	4.0	11.6	7.632	1.836	4.0	11.6	7.654	1.683
	IL2	8.4	18.3	13.059	2.139	8.4	18.3	13.074	1.956
Dependent	LS	0.0	0.842	0.288	0.263	0.0	0.80	0.272	0.257

*LS* Lodging score index or LS, *PH* plant height, IL1 internode length 1, *IL2* internode length 2, *PeD* penultimate diameter

**Table 3:** The optimal values of user-defined parameters for RF, SVR, ANN, and MLR algorithms

Classifiers used	Classifiers used
Multilinear regression (MLR)	PH, PeD, IL1, IL2
Neural network (ANN)	Learning rate = 0.2, Momentum = 0.1, Iteration = 2000, Hidden layer = 3–9–8
Random forest (RF)	K = 2, M = 4, I = 100
Support vector regression (SVR)	Kernel = rbf, Gamma = 0.004, C = 0.1

*LS* Lodging score index, *PH* plant height, *IL1* internode length 1, *IL2* internode length 2, *PeD* penultimate diameter

### Statistical analysis

Advanced statistical analysis was used to evaluate and compare the diversity between Iranian wheat accessions. Box plot was drawn using ggplot2, dplyr, and ggpubr packages in R 4.3.1 software. Correlation diagrams were also drawn using corrplot and rcolorbrewer packages in R 4.3.1 To categorize wheat accessions, cluster analysis and heat map were implemented using the gplots, dendextend, and d3heatmap packages in R 4.3.1 To reveal the distribution of wheat traits and genotypes, principal component analysis (PCA) was accomplished using the factoextra packages in R 4.3.1 Machine learning methods (ANN, SVR, and RF) were run using writexl, E1071, ithir, caret doparallel, randomforest and neuralnet packages in R 4.3.1.

## Results

### Descriptive findings

**Fig. 5:**
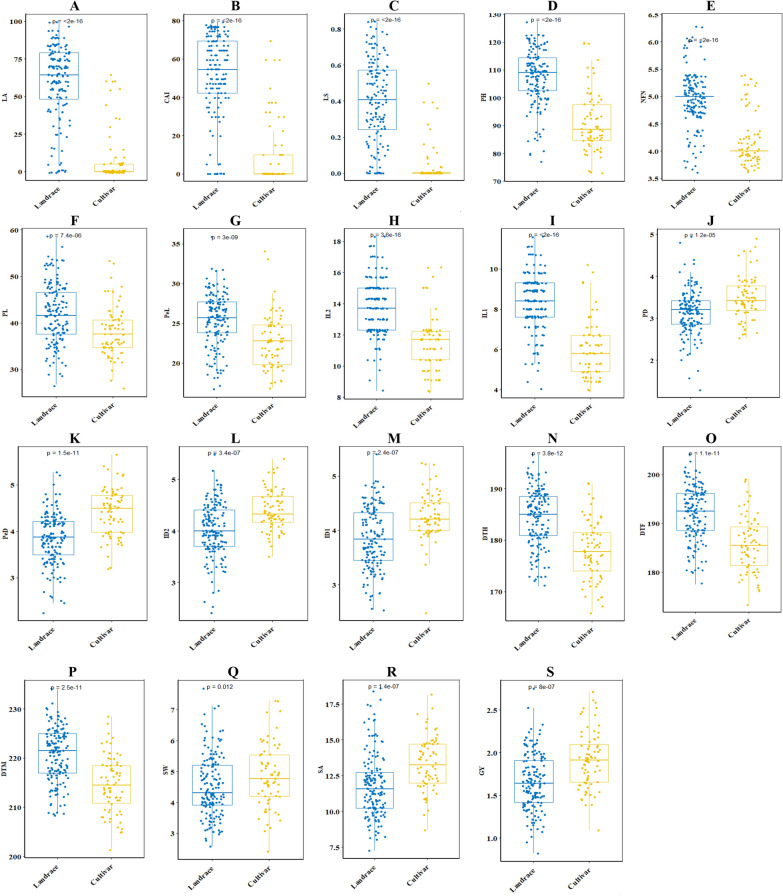
Box-plot presentation of the distribution for 19 lodging traits in Iranian wheat cultivars landraces under well-irrigated conditions. Abbreviations: Lodged area or LA (**A**), crop angle of inclination or CAI (**B**), lodging score index or LS (**C**), plant height or PH (**D**), number of nodes or NFN (**E**), peduncle length or PL (**F**), penultimate length or Pel (**G**), internode length 2 or IL2 (**H**), internode length 1 or IL1 (**I**), peduncle diameter or PD (**J**), penultimate diameter or PeD (**K**), internode diameter 2 or ID2 (**L**), internode diameter 1 or ID1 (**M**), days to heading or DTH (**N**), days to flowering or DTF (**O**), days to maturity or DTM (**P**), spike weight or SW (**Q**), spike area or SA (**R**), and grain yield or GY (**S**)

Descriptive data on lodging-related traits of wheat accessions are shown in Fig. 5. Minimum and maximum lodging area (LA), crop angle of inclination (CAI), and lodging index (LS) in cultivars and landraces were 64.4 and 100%, 69.3 and 79.2°, 0.49 and 0.84, respectively. As a result, the cultivars have a less lodging rate when compared to native landraces. Cultivars had lower height, PL, PeL, IL1, and IL2, while the stem diameter of their node was larger than native populations. Phenological traits including DTH, DTF, and DTM were lower in cultivars than landraces. Moreover, cultivars appeared superior in terms of spike weight and area, and grain yield.

### Trait correlations

**Fig. 6:**
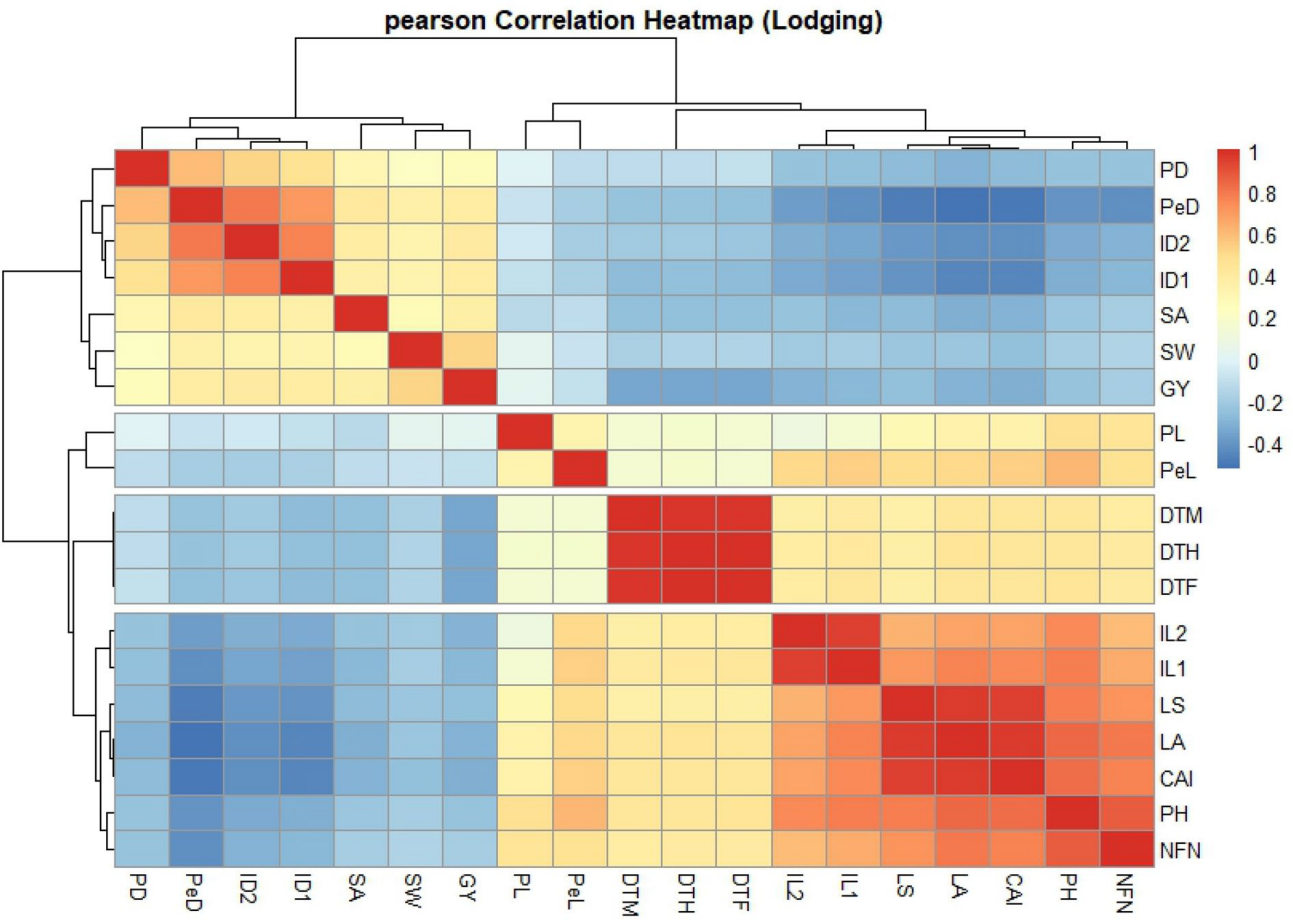
Correlation coefficients between the traits in Iranian wheat cultivars and landraces. Lodged area (*LA*), crop angle of inclination (*CAI*), lodging score index (*LS*), plant height (*PH*), number of nodes (*NFN*), peduncle length (*PL*), penultimate length (*Pel*), internode length 1 (*IL1*), internode length 2 (*IL2*), peduncle diameter (*PD*), penultimate diameter (*PeD*), internode diameter 1 (*ID1*), internode diameter 2 (*ID2*), days to heading (*DTH*), days to flowering (*DTF*), days to maturity (*DTM*), spike weight (*SW*), spike area (*SA*) and grain yield (*GY*)

The results around the correlation of lodging-related traits were shown in Fig. 6. The lodging index had the highest positive correlation with LA (r = 0.96^**^), followed by CAI (r = 0.95^**^), PH (r = 0.78^**^), NFN (r = 0.71^**^), IL1 (r = 0.70^**^), and IL2 (r = 0.63^**^). The lodging index also presented the highest negative correlation with PeD (r = − 0.48^**^), followed by ID1 (r = − 0.41^**^) and ID2 (r = − 0.40^**^). These observations reveal that the higher the lodging index, the lower the grain yield (r = − 0.26^**^).

### Principal component analysis (PCA)

**Fig. 7:**
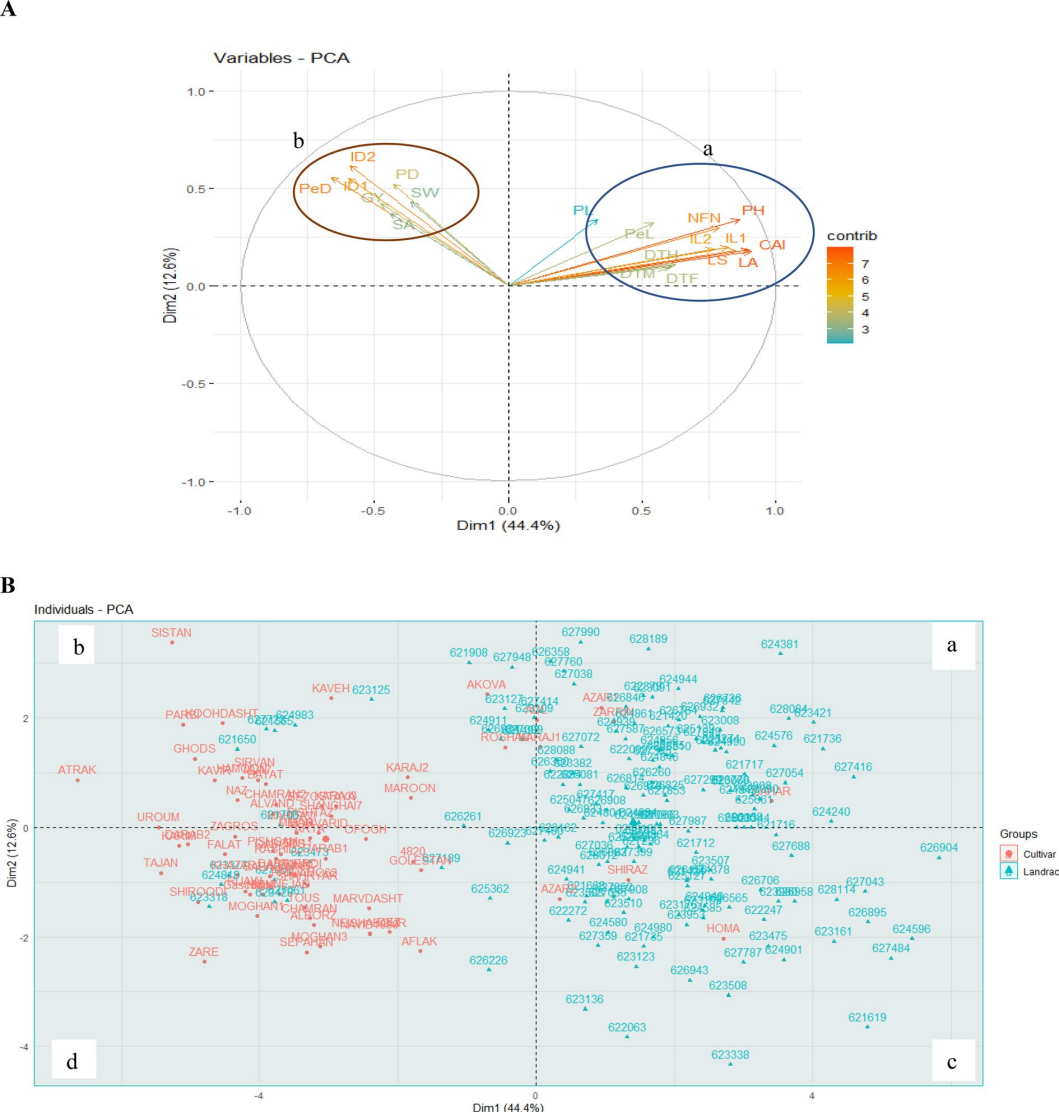
Principal component analysis of Iranian wheat landraces and cultivars. Variable biplot for the traits (**A**) and individual biplot for 228 wheat genotypes (**B**). Lodged area (*LA*), crop angle of inclination (*CAI*), lodging score index (*LS*), plant height (*PH*), number of nodes (*NFN*), peduncle length (*PL*), penultimate length (*Pel*), internode length 1 (*IL1*), internode length 2 (*IL2*), peduncle diameter (*PD*), penultimate diameter (*PeD*), internode diameter 1 (*ID1*), internode diameter 2 (*ID2*), days to heading (*DTH*), days to flowering (*DTF*), days to maturity (*DTM*), spike weight (*SW*), spike area (*SA*) and grain yield (*GY*)

The results of PCA showed that the first, second, and third components justified 44.4, 12.6, and 9.8% of the total variance, respectively. Overall, the first two components accounted for 66.8% of the total variance (Fig. 7A). According to Fig. 7A, the traits located in box *a* (LS, LA, CAI, PH, etc.) had the highest significant, positive correlation with the PC1. The traits located in the box *b* (ID1, ID2, SW, GY, etc.) had a significant, positive correlation with the PC2 and a significant, negative correlation with the PC1. Genotype-based PCA indicated that genotypes located in zone *a* had the highest lodging, genotypes located in the zone *c* had moderate to high lodging, genotypes located in zone *b* had low lodging, and genotypes located in zone *d* had without lodging (Fig. 7B). From PCA, the highest yield was recorded in the accessions located in zone *b* had than others. Genotypes located in the *b* region had a lower lodging angle (6–15°). The reason for placing the genotypes with the highest yield in this area can be due to the high spike weight (due to the thousand kernel weight and the grains number per spike), which causes the stem to have a small angle.

### Clustering

**Fig. 8:**
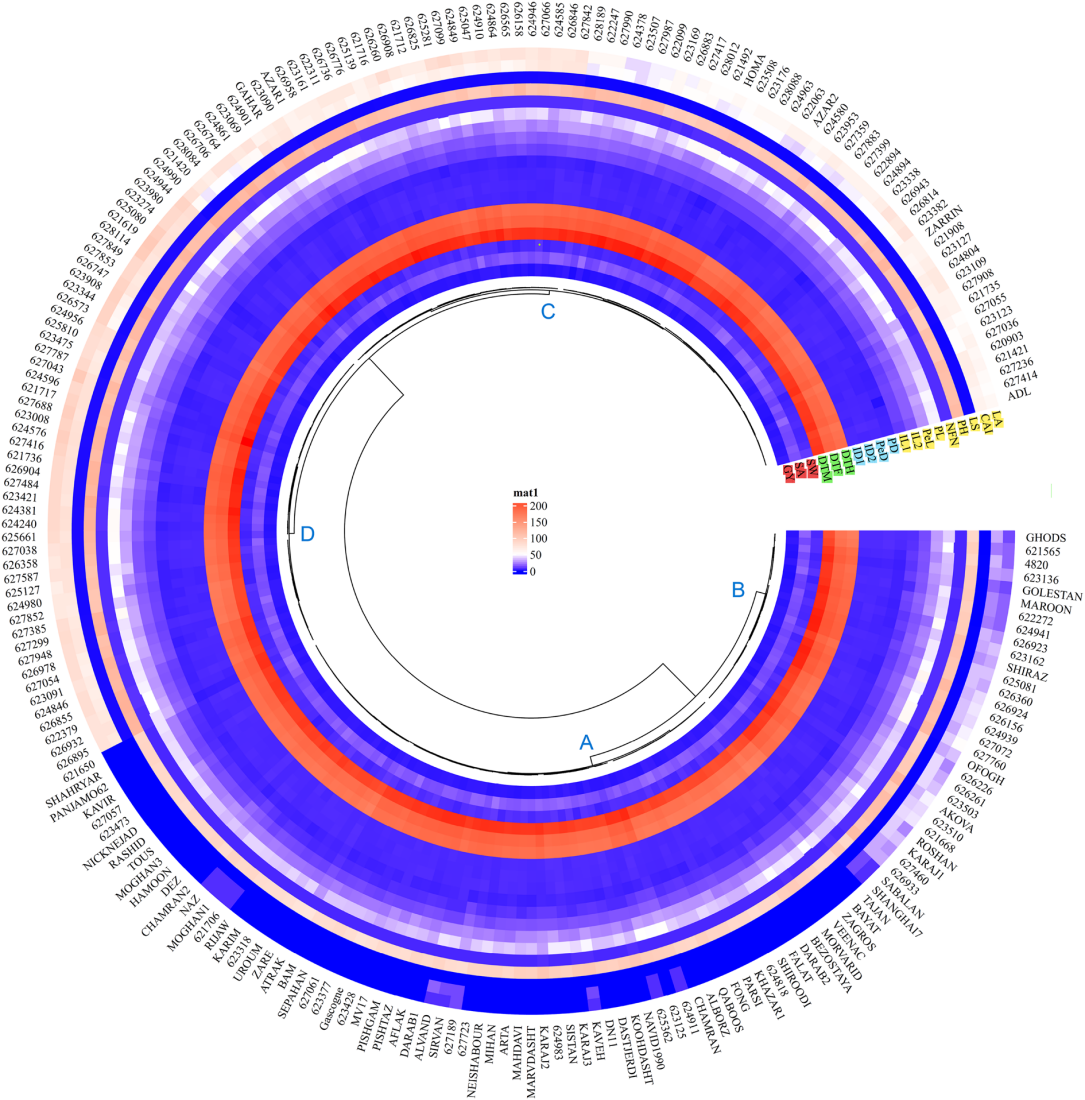
Hierarchical clustering and heatmap of Iranian wheat landraces and cultivars based on the wheat traits. Abbreviations: Lodged area (*LA*), crop angle of inclination (*CAI*), lodging score index (*LS*), plant height (*PH*), number of nodes (*NFN*), peduncle length (*PL*), penultimate length (*Pel*), internode length 1 (*IL1*), internode length 2 (*IL2*), peduncle diameter (*PD*), penultimate diameter (*PeD*), internode diameter 1 (*ID1*), internode diameter 2 (*ID2*), days to heading (*DTH*), days to flowering (*DTF*), days to maturity (*DTM*), spike weight (*SW*), spike area (*SA*) and grain yield (*GY*)

Genotypes were classified into four groups based on heat map output. The most lodging-resistant genotypes were found in group A, which had a lodging score of zero or close to zero. These accessions are the same genotypes located in zone *d* in PCA analysis. Genotypes with a lodging score between 0 and 0.15% were located in group B. In the other two groups, wheat accessions with a high lodging index score have appeared. The lodging score in group D, which includes most native populations, was the highest and ranged from 0.6 to 1 (Fig. 8). Traits were divided into four groups: group 1 including LA, CAI, LS, PH, NFN, IL1, IL2, PL, and PeL; group 2 including DTH, DTF, and DTM; group 3 including ID1, ID2, PD, and PeD; group 4 including GY, SA, and SW (Fig. 8).

### MLR analysis

**Fig. 9:**
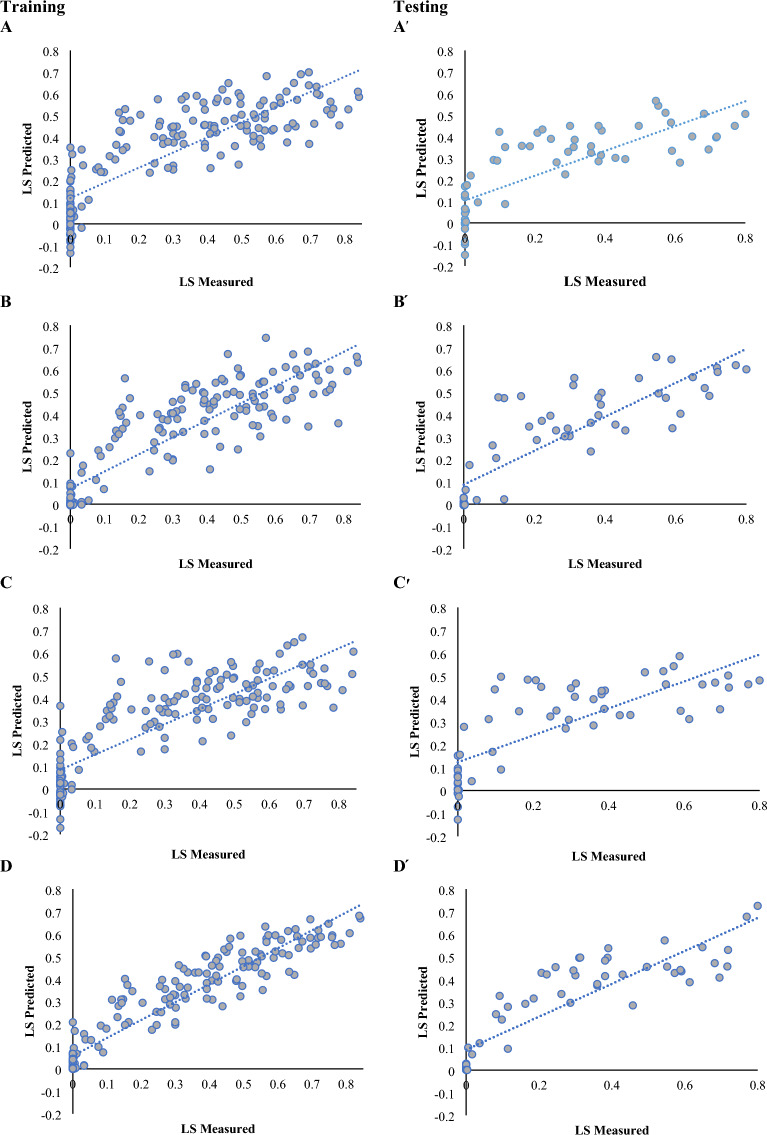
Predicted and measured lodging score index of wheat accessions using various regression methods: Scatter plot of predicted and measured lodging score index in training and testing stage of MLR (**A** and A׳), ANN (**B** and B׳), SVR (**C** and C׳) and RF (**D** and D׳)

Stepwise regression analysis was accomplished to determine the importance of the studied traits in changes in lodging index. Due to the fact that the traits of LA and CAI include the lodging index, stepwise regression analysis was performed after the removal of these traits to identify other traits affecting lodging. From the results, plant height was the first trait that entered the regression equation and alone justified about 60.6% of the changes in lodging index. PeD, IL1, and IL2 were the next traits that entered the regression equation and together with grain weight explained about 66.4% of the changes in the lodging index (Table 4). To predict the grain yield using training and testing data, stepwise regression was performed. The results showed that stepwise regression with R^2^ = 0.686 and RMSE = 0.150 for training data and with R^2^ = 0.580 and RMSE = 0.166 for testing data could predict the lodging index (Fig. 9A, A^**׳**^).

**Table 4:** Stepwise regression analysis for wheat lodging score index as the dependent variable

Step	Entered variable	Variables in model	Partial R^2^	Model R^2^	Collinearity (VIF)
1	PH	PH	0.606	0.606	1.207
2	PeD	PH, PeD	0.032	0.638	1.250
3	IL1	PH, PeD, IL1	0.016	0.654	2.588
4	IL2	PH, PeD, IL1, IL2	0.010	0.664	4.576

*LS* Lodging score index, *PH* plant height, *IL1* internode length 1, *IL2* internode length 2, *PeD* penultimate diameter

### Machine learning approaches

To predict the lodging index using input data, three different methods including RF, SVR, and ANN were compared based on two evaluation parameters R^2^ and RMSE. Artificial neural network (ANN) with R^2^ = 0.769 and RMSE = 0.126 for training data and R^2^ = 0.731 and RMSE = 0.134 for testing data could predict the amount of lodging index well (Fig. 9B, B^**׳**^). The SVR method could not have a good estimate of the lodging index and was not able to predict the genotypes that had a zero lodging index. In this method, R^2^ = 0.693 and RMSE = 0.146 were obtained for training data and R^2^ = 0.590 and RMSE = 0.163 for testing data (Fig. 9C, C^**׳**^). The RF method was able to have a good estimate of the lodging index when compared to other machine learning models, so it was able to predict accurately genotypes that had a zero lodging index. This method with R^2^ = 0.887 and RMSE = 0.091 for training data and R^2^ = 0.768 and RMSE = 0.124 for testing data was able to predict the lodging index favorably (Fig. 9D, D^**׳**^).

### Comparing MLR, ANN, SVR, and RF models for predicting LS

**Fig. 10:**
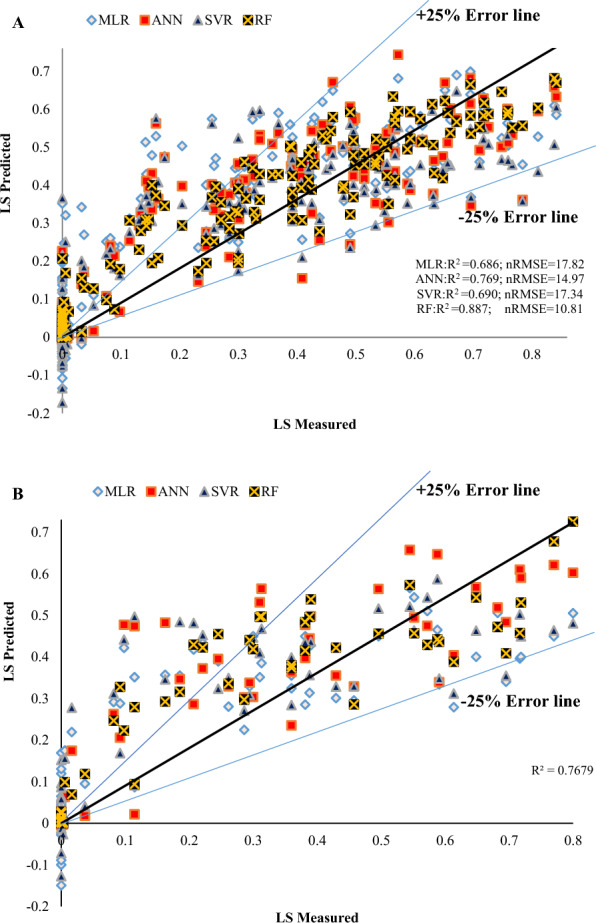
Actual vs prediction values lodging score index by using MLR, ANN, SVR and RF with training (**A**) and testing (**B**) datasets with ± 25% error line

**Fig. 11:**
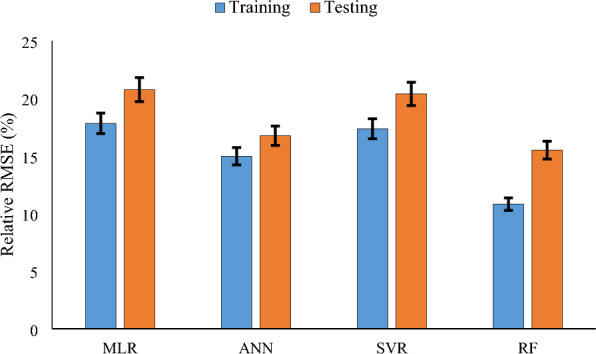
RMSE (%) outputs for lodging estimation by using MLR, ANN, SVM, and RF at the same growth stage

**Fig. 12:**
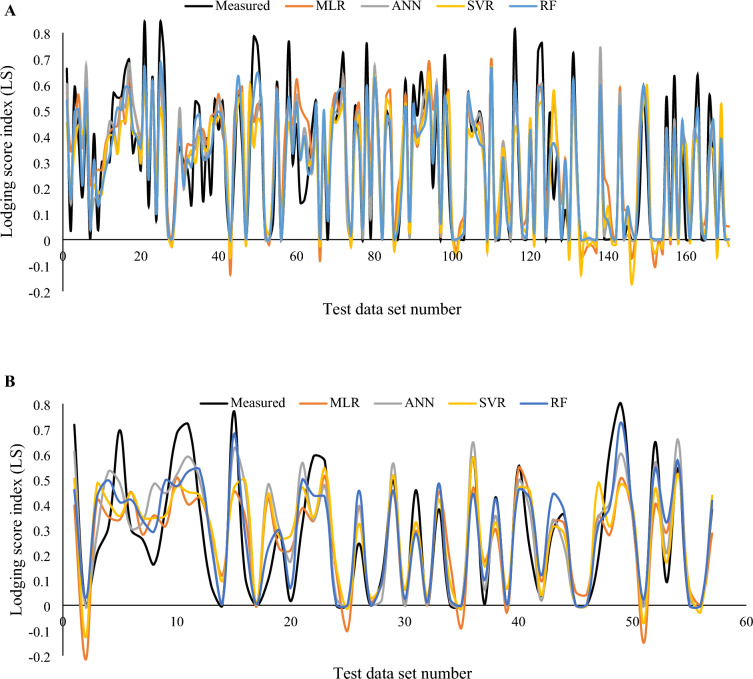
Variation in the predicted values of lodging using various regression methods in contrast to the actual value of lodging score index (A = training, B = testing)

Comparison of MLR, ANN, SVR, and RF methods showed that in all models, based on both training and testing data, the predicted values were in the range of ± 25% error line. In the RF method, more samples were found in the range of ± 25% error line. The RF method was determined as the best model compared to other methods due to the high R^2^ and low nRMSE for training and testing data. MLR, ANN, SVR, and RF models with R^2^ values including 0.686, 0.769, 0.691, and 0.887 and nRMSE values including 17.82, 14.97, 17.34, and 10.81 for training data, respectively, as well as with R^2^ values including 0.580, 0.731, 0.590, and 0.768 and nRMSE values including 20.75, 16.75, 20.37, and 15.50 for test data, respectively (Figs. 10, 11). Figure 12 shows the changes in the lodging index of actual and predicted values using training and testing datasets by MLR, ANN, SVR, and RF methods. Overall, our observations suggested that RF predicts actual data better than other algorithms (Fig. 12; Table 5).

**Table 5:** Details of parameters used for evaluating yield using MLR, ANN, SVR and RF on training and testing data sets

Models	Training			Testing		
	R^2^	RMSE	MAE	R^2^	RMSE	MAE
Multilinear regression (MLR)	0.686	0.150	0.119	0.580	0.166	0.130
Neural network (ANN)	0.769	0.126	0.089	0.731	0.134	0.095
Random forest (RF)	0.887	0.091	0.067	0.768	0.124	0.094
Support vector regression (SVR)	0.693	0.146	0.109	0.591	0.163	0.122

## Discussion

Image processing analysis has appeared as an innovative method, which permits a high number of plant morphology properties to be monitored [39]. By using this approach, you can optimize the process and make it straightforward; analyze large amounts of data at high speeds; prevent the sample from being destroyed; and analyze data at low costs [40]. Further, this approach can be used to explore possible homonyms and synonyms in a variety of assays, such as agronomic ones [41].

Diversity in Iranian wheat accessions uncovered that the cultivars have less lodging rate when compared to the native landraces due to lower DTH, DTF, and DTM, and higher stem diameter and grain yield. Stem diameter, especially in the lower internodes, explains 55% of the variance in lodging index [42], thus it is regarded as a key parameter for enhancing lodging resistance due to more lignin, cellulose, and water-soluble carbohydrates. An increase in lower internode diameter can decrease tillers per unit area and eventually grain yield [43]. Therefore, the association between grain yield and stem structure requires to be appropriately explored in wheat accessions. The relationship between phenological traits and lodging suggests that an increase in DTH, DTF, and DTM can lead to further growth, which in turn, weighs wheat down and cause lodging event [44, 45].

From trait correlations, lodging was found to be directly linked with plant height and other stem traits [46]. In facts, stem properties and their composition remarkably contribute to crop resistance to stem bending [4, 47]. As observed, the correlation between the lodging index and ID1 was slightly higher than ID1, suggesting that the first internode is more important for wheat resistance to lodging. In justifying this association, we must point out that the first internode harbors nearly twice the material strength as the second internode [48]. In addition to the irreversible bending of the stem, the displacement of the root anchorage is a critical element in lodging. Anchorage failure is influenced by low stem strength, root traits, and soil structure [49], and a weakness in any of these can contribute to lodging susceptibility. Therefore, for characterizing of wheat accessions in lodging resistance, it is highly suggested that both root and stem characteristics should be evaluated. Berry et al. [50] observed that a slight increase in root anchorage and stem strength can reduce lodging risk. In line with our observations, Tripathi et al. [42] indicated that lodging resistance negatively is associated with spike area and weight. Thus, a decrease in spike area and weight of wheat genotypes can reduce lodging risk, and these genotypes are recommended as parents for breeding programs to improve lodging resistance [48].

Previous reports already utilized various algorithms in the machine learning area for estimating biomass and related traits [22, 26–31, 51]. However, it remains unclear whether these algorithms are suitable to predict wheat lodging in the field. The current work was focused on comparing MLR with RF, ANN, and SVR for estimating lodging of Iranian wheat accessions in the field.

The RF algorithm harbored lower RMSE and higher R^2^ values than the ANN and SVR algorithms for lodging estimation, recommending that RF approach provides a precise estimation of wheat lodging. In this case, mtry determines the specific size of the subset. In comparison to SVR and ANN, this method performs fairly well [38]. Both training and testing datasets showed similar robustness to RF, and ANN showed better robustness than SVR. Random Forest models have a slightly higher generalization capability than ANN models, which behave relatively unpredictably when used with input data different from those used in training [52]. All variables are split according to the best split in the standard regression tree. Unlike this strategy, RF splits each node according to the best of a set of variables chosen randomly based on the node's location. RF achieved equivalent robustness (i.e., relative RMSE %) with ANN in both the testing and training datasets, and exhibited better robustness than SVR, as reported by Wang et al. [38]. Albeit the RF algorithm seems to be contradictory, it carries out relatively well in contrast to other machine learning models. Similarly, Wang et al. [38] achieved satisfactory findings when measuring biomass values in the field via RF. Most of the Lodging-related traits in this study are correlated. It is worth noting, RF is not susceptible to the linear association between two explanatory traits [53]. This is valuable in wheat lodging modeling since it is commonly difficult to decide which trait to remove when two (or more) traits are associated with each other [54].

Artificial neural network demonstrated weaker performance in testing than in training. This is because of the fact that RF and SVR algorithms are appropriate for a small amount of sampling data, while ANN is usually exerted on a large amount of sampling data [38]. Another cause for this is possible that the learning capability is too strong throughout the training, and therefore the model cannot reveal the hidden rules of samples finally weaken prediction capability.

This study shows wheat lodging was more accurately predicted when four traits were combined with RF regression algorithms. For the first time, we propose the use of RF regressions for lodging imaging processing. However, optimizing the modeling algorithms could improve the prediction accuracy of the method. In previous studies, different lodging parameters have been monitored at different growth stages using a single algorithm based on remotely sensed and image processing data [55]. Using non-destructive monitoring and precise modeling methods, this research contributes to the establishment of management strategies for non-destructive monitoring.

## Conclusion

Lodging remarkably decreases the quality/quantity of wheat growth and yield. The lodging index had the highest positive correlation with LA (r = 0.96**), followed by CAI (r = 0.95**), PH (r = 0.78**), NFN (r = 0.71**), IL1 (r = 0.70**), and IL2 (r = 0.63**). To estimate lodging in a non-destructive and rapid manner, various machine learning predictive algorithms were employed. In order to predict lodging in wheat, independent variables PH, PeD, IL1 and IL2 were used in model training. The findings revealed that the RF algorithm provided a more accurate estimate (R^2^ = 0.887 and RMSE = 0.091 for training data and R^2^ = 0.768 and RMSE = 0.124 for testing data) of wheat lodging. The RF algorithm was found as relatively robust as ANN and more robust than SVR.

One of the most important limitations of this research is the lack of sufficient funding to use an unmanned aerial vehicle (UAV) to take images of different genotypes of wheat on a larger scale and check the results with digital and manual imaging methods of this study.

In summary, this study provides evidence of the potential of high-resolution Image processing data in estimating CAI as a measure of lodging severity assessment, which to the best of our knowledge, has not been documented in the literature. This study proposes a new workflow pipeline for wheat lodging assessment in high-throughput plant phenotyping scenarios. It can provide important methodological reference for large-area, high-efficiency and low-cost wheat lodging monitoring research, and provide decision support for agricultural insurance and other fields.

## Data Availability

The data that support the findings of this study are available from the corresponding author, upon reasonable request.
