# Correction: Identifcation and estimation of lodging in bread wheat genotypes using machine learning predictive algorithms

**DOI:** 10.1186/s13007-024-01203-5

**Published:** 2024-06-08

**Authors:** Ehsan Rabieyan, Reza Darvishzadeh, Hadi Alipour

**Affiliations:** Department of Plant Production and Genetics, Urmia University, Urmia, Iran

**Correction: Plant Methods(2023) 19: 209**

10.1186/s13007-023-01088-w

In this article the wrong figure appeared as Figs. 6 and 8.; the figures should have appeared as shown below.

The original article has been corrected.

**Fig. 6:**
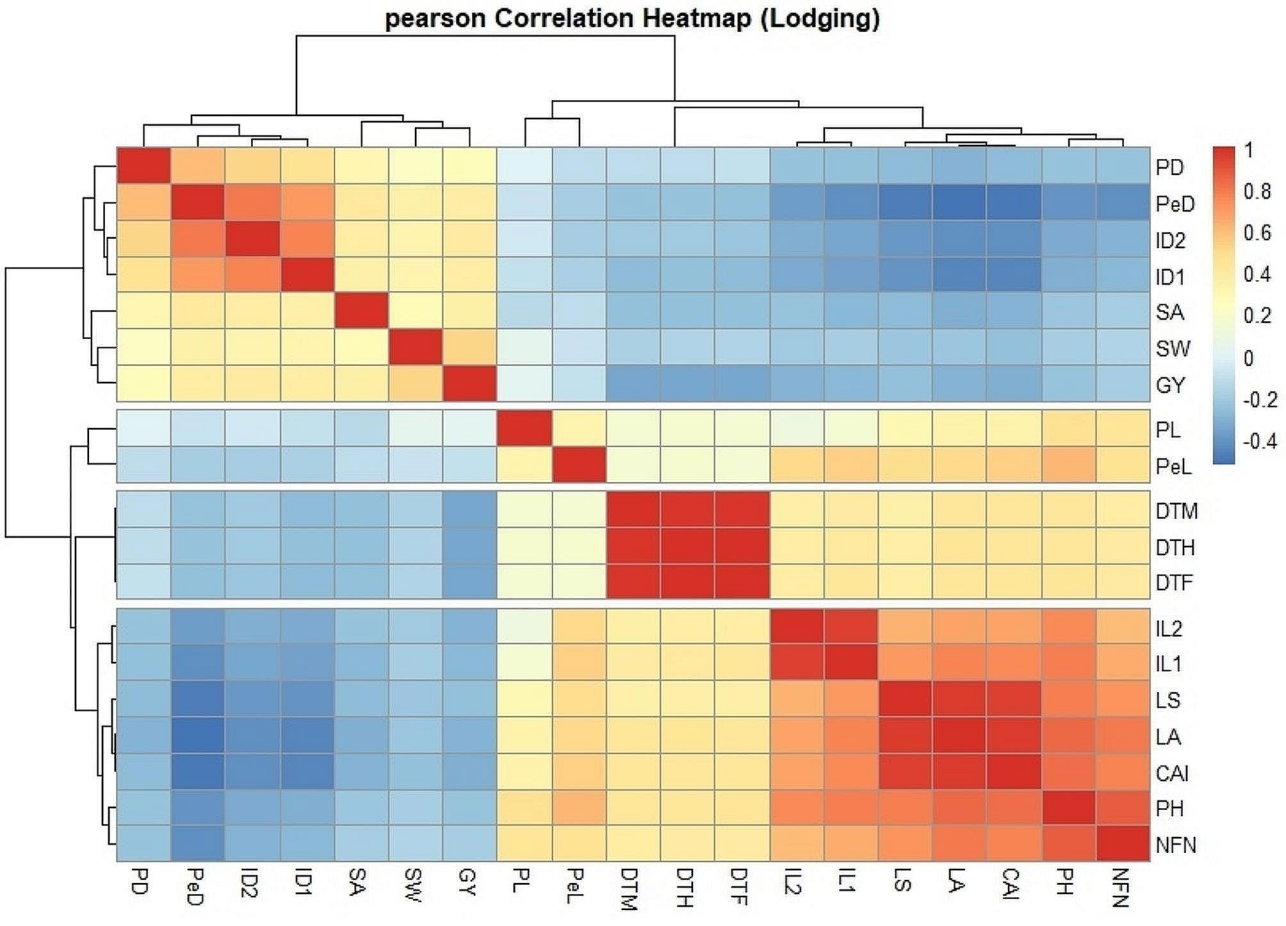
Correlation coefcients between the traits in Iranian wheat cultivars and landraces. Lodged area (LA), crop angle of inclination (*CAI*), lodging score index (*LS*), plant height (*PH*), number of nodes (*NFN*), peduncle length (*PL*), penultimate length (*Pel*), internode length 1 (*IL1*), internode length 2 (*IL2*), peduncle diameter (*PD*), penultimate diameter (*PeD*), internode diameter 1 (*ID1*), internode diameter 2 (*ID2*), days to heading (*DTH*), days to fowering (*DTF*), days to maturity (*DTM*), spike weight (*SW*), spike area (*SA*) and grain yield (*GY*)

**Fig. 8:**
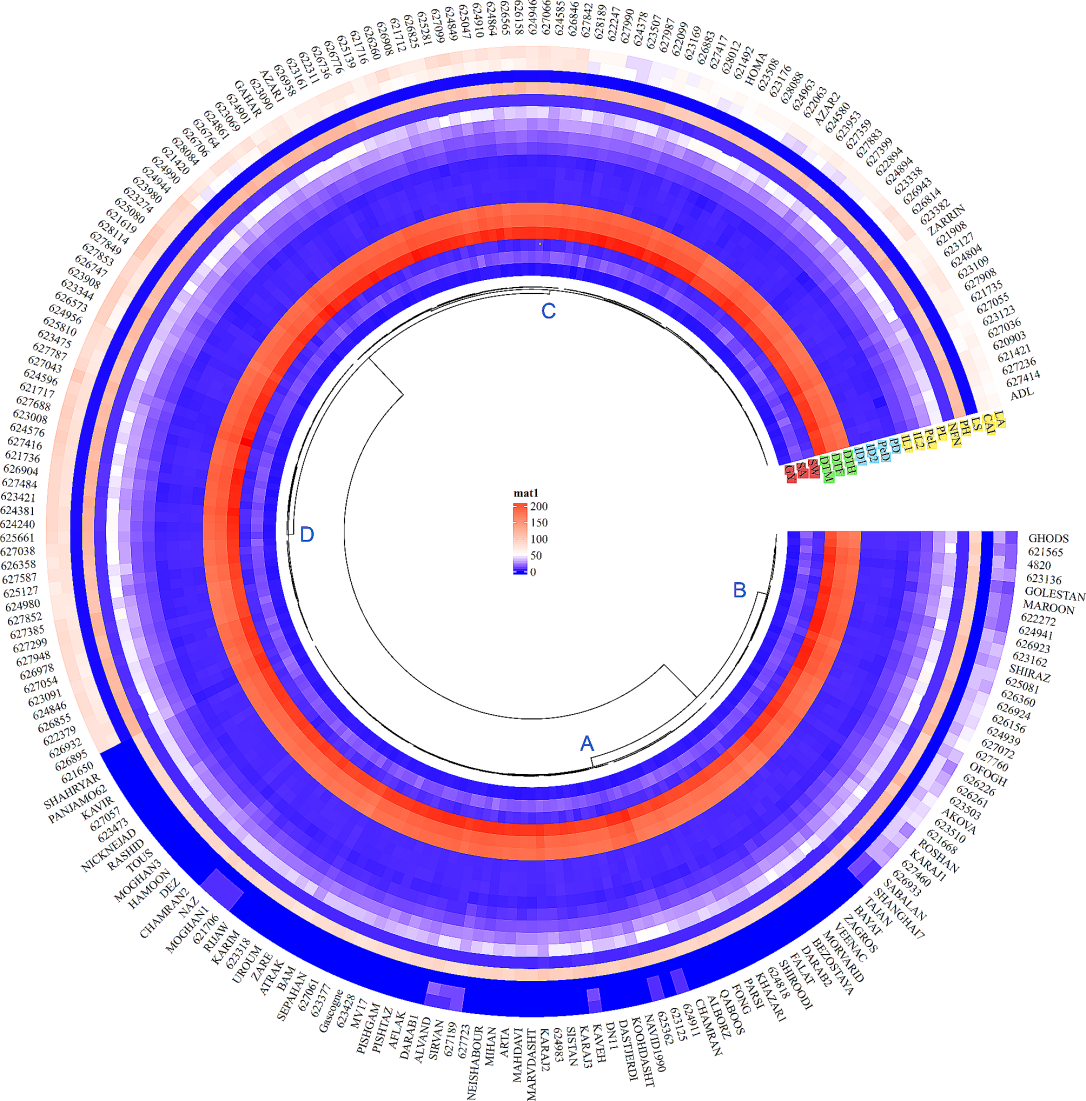
Hierarchical clustering and heatmap of Iranian wheat landraces and cultivars based on the wheat traits. Lodged area (LA), crop angle of inclination (*CAI*), lodging score index (*LS*), plant height (*PH*), number of nodes (*NFN*), peduncle length (*PL*), penultimate length (*Pel*), internode length 1 (*IL1*), internode length 2 (*IL2*), peduncle diameter (*PD*), penultimate diameter (*PeD*), internode diameter 1 (*ID1*), internode diameter 2 (*ID2*), days to heading (*DTH*), days to fowering (*DTF*), days to maturity (*DTM*), spike weight (*SW*), spike area (*SA*) and grain yield (*GY*)

